# Case Report: Kryptonite—A Rare Case of Left-Sided Bilothorax in a Sickle Cell Patient

**DOI:** 10.1155/2019/8658343

**Published:** 2019-06-18

**Authors:** Vikas D. Reddy, Anas Al-Khateeb, Muhammad Hussain, Varun Patel, Muqueet Kadri, Rutwik Patel, Siva Prasad Maruboyina, Richard A. Miller, Joseph R. DePasquale

**Affiliations:** ^1^Department of Internal Medicine, Saint Michael's Medical Center, Newark, NJ, USA; ^2^Department of Pulmonary and Critical Care, Saint Michael's Medical Center, Newark, NJ, USA; ^3^Department of Gastroenterology, Saint Michael's Medical Center, Newark, NJ, USA

## Abstract

Bilothorax is a rare cause of an exudative pleural effusion. The diagnosis is confirmed by a pleural fluid to serum bilirubin ratio of greater than 1. Typically, bilothorax presents as a right-sided effusion due to its proximity to the liver and biliary system. Herein, we present a case of isolated left-sided bilothorax in a 43-year-old female admitted with sickle cell crisis. Only one other case of isolated spontaneous left-sided bilothorax has been described in the literature. A thoracentesis performed on admission demonstrated greenish fluid and bilothorax was suspected, with a pleural fluid to serum bilirubin ratio greater than 1 confirming the diagnosis. A magnetic resonance cholangiopancreatography (MRCP) showed an abnormal 90-degree acute angulation in the mid-to-distal common bile duct with proximal common bile duct and intrahepatic bile ducts dilation. This was further confirmed with an endoscopic retrograde cholangiopancreatography (ERCP), which did not reveal any extravasation of contrast into the left pleural space. Ultimately, despite the use of various modalities, no definitive cause of bilothorax was identified. Postthoracentesis imaging revealed evidence of fibrothorax, a direct and permanent complication of bilothorax. The presence of an isolated left-sided bilothorax, along with the lack of a confirmed etiology, makes this case unique.

## 1. Case

A 43-year-old African American female with sickle cell disease presented with two days of severe generalized pain and was found to be in sickle cell crisis. Her past medical history was also significant for asthma, hypothyroidism, and secondary hemochromatosis due to multiple previous blood transfusions. She denied fever, cough, nausea, and vomiting but reported malaise and generalized fatigue. She also had an unintentional weight loss of twenty pounds over the last four months. Surgical history included a laparoscopic cholecystectomy three years prior, with no known complications. Although she had several hospitalizations for sickle cell crises in the last two years, there was no recent history of trauma or invasive procedures. She denied drinking alcohol or smoking tobacco but did admit to occasionally smoking marijuana. Her medications included hydromorphone, tramadol, levothyroxine, folic acid, and an albuterol inhaler as needed. She also received iron chelation therapy as an outpatient.

Vital signs showed temperature 98.1 Fahrenheit (F), pulse 97 beats per minute (bpm), respiratory rate of 17, blood pressure (BP) of 123/74 mmHg, and oxygen saturation of 100% breathing ambient air. On physical examination, the patient was markedly cachectic and appeared to be in mild distress from pain, however was able to speak in complete sentences. Scleral icterus was present, along with a grade 3/6 systolic flow murmur best heard at the left 5th intercostal space. On pulmonary examination, auscultation revealed decreased breath sounds and dullness to percussion over the left lung base. Abdominal exam was significant for diffuse tenderness to palpation and mild hepatomegaly, but no rebound or rigidity was appreciated. There was no evidence of active bleeding and no neurological deficits were noted.

**Figure 1:**
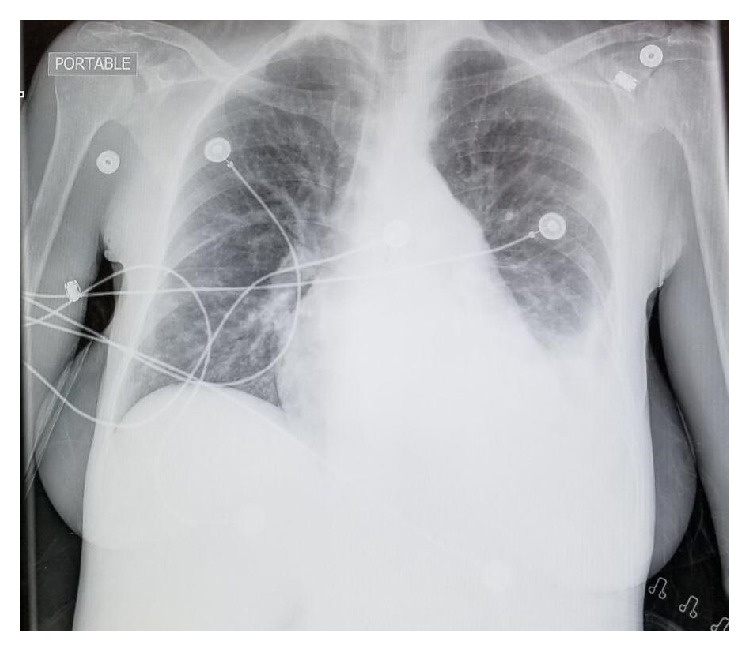
Initial chest X-ray demonstrating an isolated left-sided pleural effusion.

The reticulocyte count was elevated at 19%, serum LDH was elevated at 279, haptoglobin was undetectable, and hemoglobin was decreased from her baseline at 6.1 g/dL. Coombs test was negative. Her initial chemistry panel was abnormal with a total bilirubin of 3.0 mg/dL, direct bilirubin level of 2.06 mg/dL, AST 61 U/L, ALT 33 U/L, ALP 570 U/L, GGT 763 U/L, and albumin 2.5 g/dL. There were no abnormalities of renal function or electrolytes. Her INR was 1.3 and ferritin 7,623 ng/mL. WBC count and platelets were elevated at 13.7 cells/*μ*L and 415 cells/*μ*L, respectively. Ultrasound of the abdomen showed hepatomegaly with a span of 20 cm and mild dilatation of the pancreatic duct, measuring 4 mm. A chest X-ray revealed an isolated large left pleural effusion (Figure 1).

**Figure 2:**
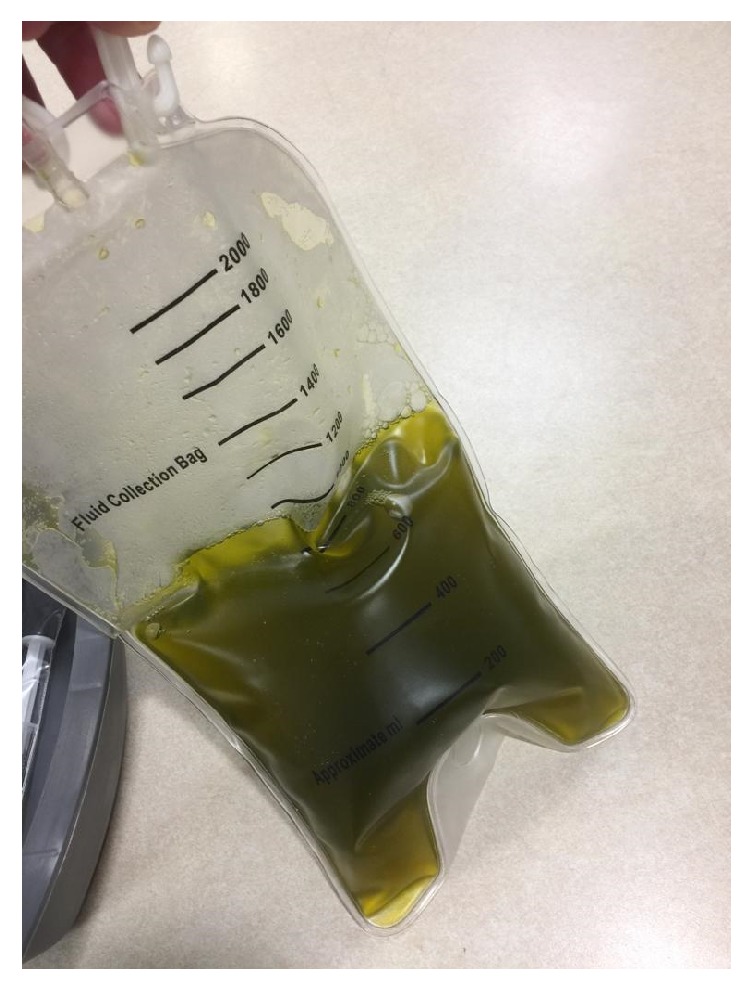
A thoracentesis yielded 900 cc of exudative bilious fluid.

In addition to aggressive intravenous hydration, iron chelation therapy was initiated along with cautious blood transfusion. Antibiotics were empirically started. Thoracentesis was performed revealing 900 cc of green bilious-appearing fluid (Figure 2) with pleural fluid analysis (PFA) showing an elevated pleural bilirubin (3 mg/dL) to serum bilirubin (2.2 mg/dL) ratio greater than 1, confirming the diagnosis of an isolated left-sided bilothorax (Table 1). Pleural fluid studies also showed WBC 676 cells/*μ*L with a lymphocytic predominance (59%), glucose 97 mg/dL, and protein of 8.2 mg/dL. Pleural fluid cytology, gram stain, and culture were negative for infection.

**Figure 3:**
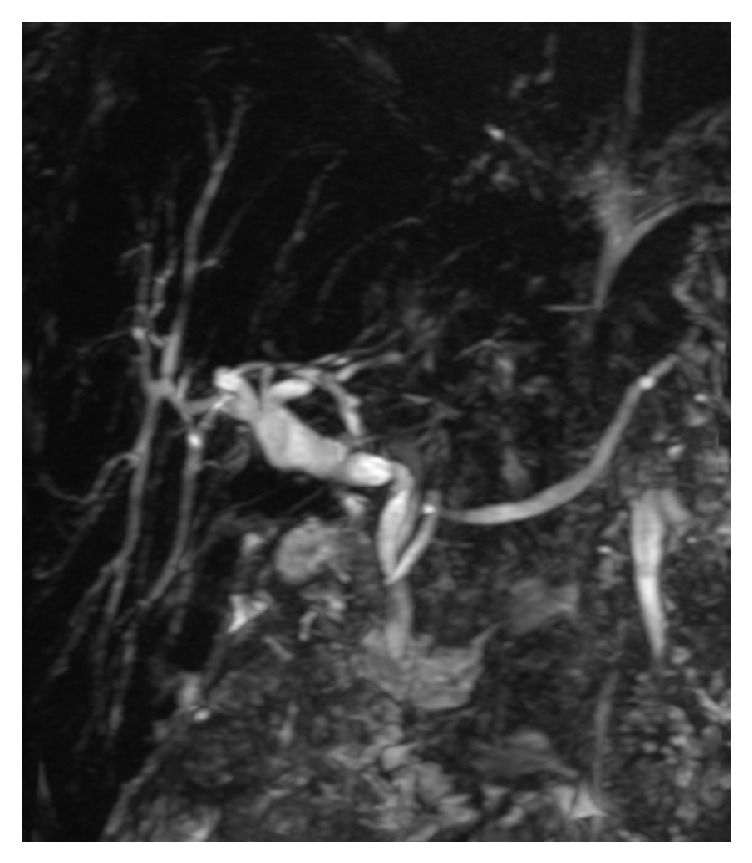
Magnetic resonance cholangiopancreatography (MRCP) showing an acute angle in the mid-common bile duct with upstream dilatation.

**Figure 4:**
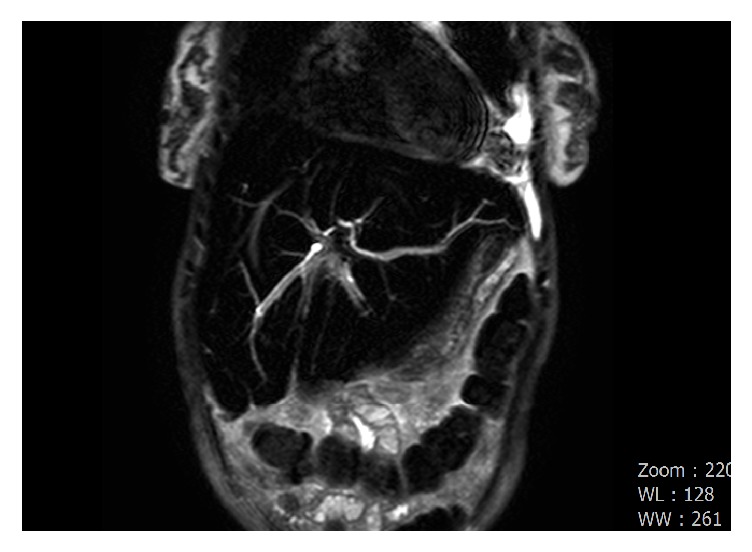
Coronal image of an MRCP showing an isolated left intrahepatic duct extending through the left hepatic lobe to the left costophrenic angle.

A magnetic resonance cholangiopancreatography (MRCP) showed iron deposition in the liver and bone marrow as well as considerable dilatation of the intrahepatic bile ducts. An abnormal 90-degree acute angulation in the mid-to-distal common bile duct (CBD) was found 2.7 cm from the ampulla of Vater, with proximal CBD and intrahepatic duct dilation. The distal CBD measured 8 mm (Figure 3). An isolated left intrahepatic duct was seen extending to the distal margin of the left hepatic lobe; however, no clear fistulous connection was seen with the left pleural space (Figure 4). No evidence of choledocholithiasis or pancreatic mass was seen.

**Figure 5:**
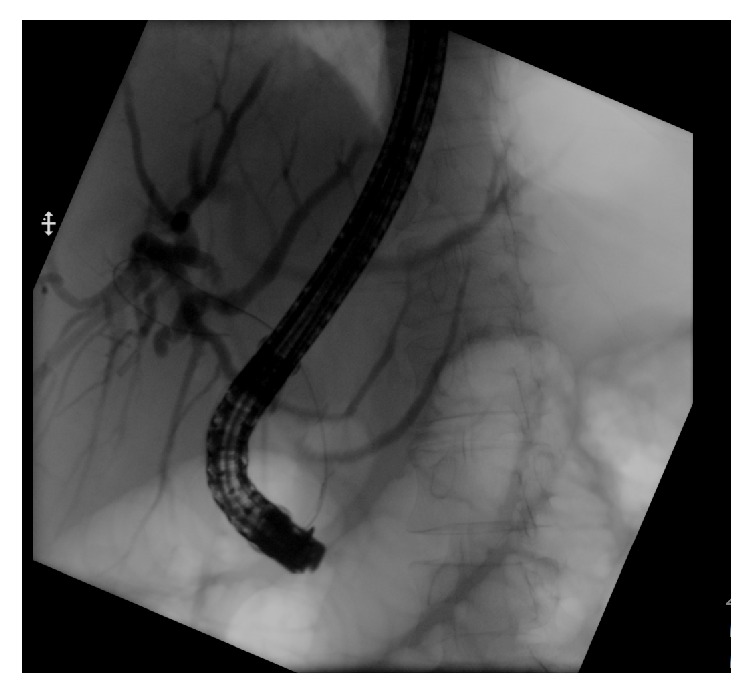
Endoscopic retrograde cholangiopancreatography (ERCP) fluoroscopic image showing dilated intrahepatic ducts with absence of filling on the common bile duct (evidenced by the guidewire).

A hepatobiliary scintigraphy scan (HIDA) scan was negative for biliary leak. An endoscopic retrograde cholangiopancreatography (ERCP) showed prominent intrahepatic biliary ducts, however the mid-to-distal common bile duct was poorly visualized below the sharp angulation (Figure 5). No stricture or leak was seen on multiple injections. A 10 French x 7 cm plastic stent was successfully placed crossing the sharp angulation in the CBD to eliminate the pressure differential in the duct and provide adequate drainage. Given the improvement in symptoms, decortication was deferred during the admission. The patient was scheduled to follow up within two weeks after discharge to schedule a CT of the chest and repeat ERCP with stent removal if the bilothorax had resolved.

The patient was subsequently lost to follow-up despite multiple attempts to reach the patient. Three months after the initial presentation, the patient presented after several syncopal episodes associated with watery diarrhea. She was found to have sickle cell crisis, profound hypoglycemia, and signs of septic shock complicated by disseminated intravascular coagulation (DIC). Initial vital signs showed a temperature 100.2 F, pulse 120 bpm, respiratory rate of 24, BP of 63/28 mmHg, and oxygen saturation of 82% breathing ambient air. Her chemistry panel revealed severe metabolic acidosis and acute renal failure. Her hemoglobin on presentation was 3.9 g/dL, white blood cell 17,000 cells/*μ*L, and platelet level of 56 cells/*μ*L. She developed multiorgan failure along with an initial lactic acid level of 18.2 mmoL/L, procalcitonin elevated at 165.35 ng/mL, and other markers indicating DIC. Pan CT-imaging confirmed bilateral lobar consolidation suspicious for pneumonia and thickening of the jejunum and ascending colon consistent with enterocolitis. On CT abdomen, there was resolution of her previous biliary ductal dilatation with an appropriately positioned common bile duct stent and no recurrence of the bilothorax.

She required intubation and was immediately started on intravenous fluids, pressor support, and broad-spectrum antibiotics. Despite aggressive efforts, the patient sustained a cardiopulmonary arrest and unfortunately passed. Septic workup confirmed bacteremia with blood cultures growing* Streptococcus pneumoniae*. Functional asplenia and a diminished pulmonary reserve from permanent fibrothorax (due to her previous bilothorax) rendered the patient more susceptible to this encapsulated bacterium.

## 2. Discussion

Bilothorax, or cholethorax, is a rare and potentially dangerous entity defined by the presence of bile in the pleural fluid. Bilothorax was first described by Williams, et. al in a case in which it developed secondary to a biliopleural fistula formation caused by blunt trauma to the liver [1]. In medical literature, notable causes of bilothorax have been traumatic injury such as stab wounds, gunshots or blunt trauma, followed by procedures such as percutaneous transhepatic biliary drainage. Other etiologies of bilothorax have consisted of biliary obstruction, hepatic and subphrenic abscesses, hydatid cystic disease (echinococcosis), percutaneous cholangiography, cholecystopleural fistula, post-ERCP complications, percutaneous liver biopsy, and gastropleural fistula formation after gastric perforation with nasogastric tube [2].

In this case, the patient presented with sickle cell crisis with jaundice and was found to have an incidental finding of a left-sided pleural effusion. A left-sided thoracentesis yielded greenish fluid with an elevated pleural fluid to serum bilirubin ratio greater than 1, which is diagnostic for bilothorax. Further investigations including a HIDA scan did not demonstrate direct leakage from the biliary tree to the pleural space. Therefore, an ERCP was performed for both diagnostic and therapeutic intent which confirmed the angulation and dilatation of the biliary tree, however no stricture, mass lesion or stones were found. A stent was placed to decompress the dilated left intrahepatic biliary tree. The management of bilothorax includes drainage of the pleural effusion and treatment of the underlying cause, if identified. In cases where a fistula is identified, spontaneous closure of the fistula rarely occurs. Bile in an enclosed area is a good medium for the growth of bacteria. Emphysematous bilious pleural effusions often involve organisms from the gastrointestinal tract such as* Escherichia coli*,* Enterobacter spp.*, and even* Staphylococcus aureus* [3]. In our case, the CT scan of the chest showed bilateral lobar consolidation coupled with pneumococcal bacteremia which was most consistent with a pulmonary etiology of infection. To confirm clearance of the bilothorax, an autopsy would have been beneficial. Unfortunately, the family refused.

Nearly all cases of bilothorax reported in medical literature have been right-sided and with a clear etiology including in most cases a fistulous connection. The fistula is thought to be formed following trauma with the development of a biloma and its rupture into the pleural cavity, a process that can take weeks to develop [4]. Another mechanism is the passage of bile from the peritoneum to the pleural cavity via a defect in the diaphragm, in the same way ascitic fluid results in hydrothorax formation [5].

There are only a few cases of left-sided bilothorax reported in the literature. One similar case with a left-sided bilothorax was attributed to a cholangiocarcinoma causing intrahepatic dilatation and subsequent fistulous connection [6]. Other cases have involved trauma from a car accident and nasogastric tube placement complicated by the formation of a gastropleural fistula due to stomach perforation [7, 8]. Left-sided bilothorax in a patient with no identifiable etiology or biliary leak is rarely found. Only one other case of spontaneous left-sided bilothorax formation in a patient with acute pancreatitis as the possible etiology has been reported in the literature [9].

In our case, given the absence of a definite fistulous connection and absence of ascites in the patient, we postulate that the acute angulation found in the mid-to-distal common bile duct (CBD) played a role from her cholecystectomy three years prior to her presentation - although there were no documented immediate complications from the surgery. The increased pressure above the acute angulation likely created the aberrant left intrahepatic duct near the left hemidiaphragm. This may have led to microscopic connections to the left pleural space, creating a path for bile to accumulate within the negative pressure thorax. Given this hypothesis, ERCP and placement of a 10 French x 7 cm plastic biliary stent was intended to decompress the left biliary tree to reduce the upstream pressure within the bile duct and repair the presumed small leak. The absence of bilothorax on readmission following stent placement supports this theory. The presence of thickened pleura and fibrothorax on imaging after thoracentesis confirms that the leak was chronic, further supporting our hypothesis.

Spontaneous bilothorax of the left pleural space is a rare event; this case is the second case reported in the literature. Even though this is a rare event, suspicion for bilothorax should be considered in any patients with known biliary injury and also when the appearance of the fluid suggests a biliary origin. Importantly, bile in the pleural space has a tendency to promote infection and ultimately leads to the formation of an exudative pleural effusion [10]. Therefore, diagnosis must be confirmed with measurement of the pleural to serum bilirubin ratio. The mainstay of treatment of bilothorax includes drainage of the effusion and correction of any identifiable underlying causes.

## Figures and Tables

**Table 1:** Pleural fluid analysis (PFA).

Pleural Fluid Analysis	Value
Color	Green
Type	Exudate
Total Bilirubin P/S Ratio	1.36
Total Pleural Bilirubin	3.0 mg/dL
pH	7.626
RBCs	749/hpf
WBCs	673/hpf
Neutrophils	24%
Lymphocytes	59%
Monocytes	5%
Total LDH P/S Ratio	0.57
Glucose	97 mg/dL
LDH	160 IU/L
Protein	8.2 mg/dL
Gram Stain	Negative
Culture	Negative
